# Approach to Cerebrospinal Fluid (CSF) Biomarker Discovery and Evaluation in HIV Infection

**DOI:** 10.1007/s11481-013-9491-3

**Published:** 2013-08-13

**Authors:** Richard W. Price, Julia Peterson, Dietmar Fuchs, Thomas E. Angel, Henrik Zetterberg, Lars Hagberg, Serena Spudich, Richard D. Smith, Jon M. Jacobs, Joseph N. Brown, Magnus Gisslen

**Affiliations:** 1 Department of Neurology, University of California San Francisco, San Francisco General Hospital, Bldg 1 Room 101, Potrero Avenue, Box 0870 1001, San Francisco, CA 94110 USA; 2 Division of Biological Chemistry, Innsbruck Medical University, Innsbruck, Austria; 3 Biological Sciences Division, Pacific Northwest National Laboratories, Richland, WA USA; 4 Institute of Neuroscience and Physiology, Department of Psychiatry and Neurochemistry, the Sahlgrenska Academy at the University of Gothenburg, Gothenburg, Sweden and Institute of Neurology, Queen Square, London, UK; 5 Department of Infectious Diseases, Sahlgrenska Academy at the University of Gothenburg, Gothenburg, Sweden; 6 Yale University, New Haven, CT USA

**Keywords:** HIV, Cerebrospinal fluid, CSF, Nervous system, Biomarkers, Neopterin, Neurofilament, Proteomics, Inflammation

## Abstract

Central nervous system (CNS) infection is a nearly universal facet of systemic HIV infection that varies in character and neurological consequences. While clinical staging and neuropsychological test performance have been helpful in evaluating patients, cerebrospinal fluid (CSF) biomarkers present a valuable and objective approach to more accurate diagnosis, assessment of treatment effects and understanding of evolving pathobiology. We review some lessons from our recent experience with CSF biomarker studies. We have used two approaches to biomarker analysis: targeted, hypothesis-driven and non-targeted exploratory discovery methods. We illustrate the first with data from a cross-sectional study of defined subject groups across the spectrum of systemic and CNS disease progression and the second with a longitudinal study of the CSF proteome in subjects initiating antiretroviral treatment. Both approaches can be useful and, indeed, complementary. The first is helpful in assessing known or hypothesized biomarkers while the second can identify novel biomarkers and point to broad interactions in pathogenesis. Common to both is the need for well-defined samples and subjects that span a spectrum of biological activity and biomarker concentrations. Previously-defined *guide* biomarkers of CNS infection, inflammation and neural injury are useful in categorizing samples for analysis and providing critical biological context for biomarker discovery studies. CSF biomarkers represent an underutilized but valuable approach to understanding the interactions of HIV and the CNS and to more objective diagnosis and assessment of disease activity. Both hypothesis-based and discovery methods can be useful in advancing the definition and use of these biomarkers.

## Introduction

Infection of the central nervous system (CNS) begins during primary systemic infection and continues throughout its untreated course (Ellis et al. 1997; McArthur et al. 1997; Gisslen et al. 1999; Spudich et al. 2005b; Valcour et al. 2012). While seemingly innocent over much of this course and indeed clinically silent despite detectable HIV RNA and an inflammatory response in the cerebrospinal fluid (CSF), this infection may nevertheless impact brain function. Most notably, in some patients CNS infection evolves into a more invasive encephalitic form that presents clinically as HIV-associated dementia (HAD) (Navia et al. 1986; Price et al. 1988). The detailed characteristics and pathogenesis of this shift from ‘benign’ meningitis to devastating encephalitis remain poorly defined, though changes in both the viral pathogen and host immune responses likely contribute in concert (Schnell et al. 2011; Arrildt et al. 2012). Fortunately, HAD can now be largely prevented by combination antiretroviral therapy (ART), and as a result its incidence has diminished markedly in the developed world where ART is widely available (d’Arminio Monforte et al. 2004; Bhaskaran et al. 2008; Lescure et al. 2011). In these regions HAD now manifests almost exclusively in late presenters with advanced immunodeficiency. Additionally, ART can arrest progression and variably reverse the neurological dysfunction of HAD (Sidtis et al. 1993; Tozzi et al. 1999).

However, despite this success, a number of recent reports have documented impairment of neuropsychological testing performance in treated patients, with or without clear symptoms, and have suggested that milder, though still important, forms of neurological dysfunction in HIV patients remain common even in the face of systemic viral suppression (Tozzi et al. 2007; Heaton et al. 2010, 2011; Simioni et al. 2010; Smurzynski et al. 2011). These observations not only challenge the effectiveness of current therapies in preventing and treating CNS HIV infection and related CNS injury, but point to the difficulty in defining the nature and activity of the underlying disease processes using available clinical evaluation methods. Disease definitions rely chiefly on staging severity (Price and Brew 1988; 1991) and particularly on the extent of ‘impairment’ in neuropsychological test performance (Antinori et al. 2007). These definitions do not directly take into account either the cause (beyond the context of HIV infection) or the ongoing state of disease activity. This can present a particular problem in the treated patient in whom it is important to distinguish the cumulative effects of past CNS damage from the impact of ongoing injury.

The nearly universal presence of HIV in CSF whether or not overt CNS disease is present, the imprecision of phenotypic disease recognition even when extended to more quantitative measures by neuropsychological test biometrics, and the frequency of confounding background conditions all call for better objective assessments of infection and disease. These pathogenetic and clinical needs, in turn, point to the potential utility of objective, laboratory-based biomarkers in overcoming the shortcomings of purely clinical definitions of HIV-related CNS injury (Price et al. 2007).

While several types of biomarkers might help to address these issues, CSF biomarkers are likely to be among the most useful and are therefore the focus of this review which draws on our recent experience in biomarker evaluation and discovery. It describes some of the lessons we have learned in the process of these studies. After a brief introduction describing a disease framework that organizes our approach, we discuss the rationale for focusing on CSF and the potential applications of biomarkers that justify this attention. We then describe examples of two different approaches to CSF biomarkers, one hypothesis-driven and the other untargeted and exploratory.

## Disease framework for approaching CSF biomarkers

**Fig. 1:**
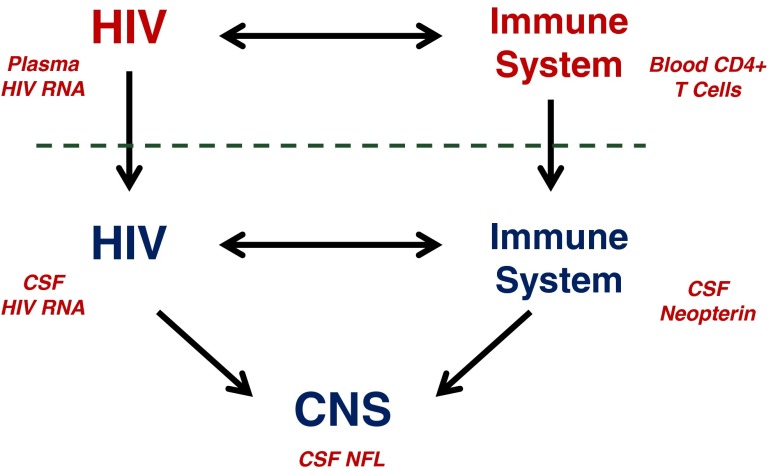
Schematic diagram of salient systemic and CNS HIV disease components. CNS HIV infection and immune responses initially are extensions across the blood–brain and blood-CSF barriers (*horizontal dashed line*) of their systemic counterparts, though with variable selection and local evolution depending on the stages of disease. Within the nervous system, both the virus and immune responses may impact the CNS and its function in the depicted *triangle*. In the small font are examples of biomarkers for each of the main pathogenic components. Plasma HIV RNA and blood CD4 cells in systemic disease have proved to be essential biomarkers in systemic management, while CSF HIV RNA, neopterin and NFL have served as principal guide biomarkers in our studies of CNS infection and injury

Figure 1 presents a simplified framework for classification of biomarkers of CNS HIV infection and disease in relation to their systemic counterparts. The top of the figure diagrams the systemic interaction of HIV and the host immune system: infection evolves over time with selection and expansion of viral populations in concert with changes in the immune system that exhibits both progressive deficiency and broad activation (Hunt 2012). These interactions eventually lead to an array of systemic diseases, including some more directly related to the virus (e.g., HIV-related nephropathy), but more frequently to opportunistic infections secondary to immunodeficiency, including those that define AIDS (http://www.aidsinfo.nih.gov/guidelines/html/4/adult-and-adolescent-oi-prevention-and-treatment-guidelines/0). Immune activation is critical to general systemic progression and also contributes to organ injuries from a variety of ‘nonAIDS-related’ diseases (El-Sadr et al. 2006, 2008; Phillips et al. 2008). Importantly, these systemic interactions can be monitored using blood biomarkers of these two disease components. Thus, magnitude of systemic infection is followed by measuring plasma HIV RNA levels that helps in predicting the rate of disease progression, while the blood CD4+ T cell count provides an index of the cumulative damage to the immune system and the vulnerability to opportunistic diseases (Mellors et al. 1997). Additional soluble and lymphocyte phenotype markers can also assess systemic immune activation and predict disease progression (Lyles et al. 1999; Giorgi et al. 2002; Neaton et al. 2010). These biomarkers have had a profound effect on the advances in clinical management and therapeutics of HIV infection; indeed, it is difficult to imagine the remarkable progress in this condition without these biomarker tools in both clinical trials and individual patient management. While more difficult to evaluate, CSF biomarkers have the potential for similar impact on management of CNS HIV infection and resultant disease.

As indicated by the vertical arrows crossing the dotted line representing the blood–brain and blood-CSF barriers in Fig. 1, CNS HIV infection and local immune-inflammatory responses originate with selective transfer from the blood into the CNS of both infection (including both infected cells and major cell targets) and systemic immune reactions. Subsequently, local infection and immune-inflammatory reactions can evolve further and impact nervous system function and integrity, thus forming a pathogenic triangle with two main interacting agonists and a target. CSF biomarkers can assess each of these three disease components in order to characterize their evolution in particular disease states and within individual patients.

We have selected a representative from each of these components as CSF *guide biomarkers* to incorporate into each of our studies: CSF HIV RNA, neopterin and neurofilament light chain (NFL) concentrations. These serve as indicators or vectors of these pathogenetic components; other terms that have been used for these types of biomarkers are *orthogonal* biomarkers and *endophenotypes* (Cruchaga et al. 2010; Angel et al. 2012). Table 1 lists these three biomarkers along with other examples from our own previous and ongoing work. Blood-brain barrier and endothelial injury might be separately classified, but for simplicity they have been included in the immune-inflammatory group.

**Table 1:** Examples of defined CSF biomarkers examined in our studies of HIV

Class of CSF biomarker	Cardinal guide biomarkers	Other biomarker examples
Viral	HIV RNA	Genetic compartmentalization, T-cell/macrophage tropism, coreceptor utilization, drug resistance
Immune	Neopterin	CCL2 (MCP-1), CXCL10 (IP-10), β2M, TNFα, sCD14, sCD163, sVCAM-1, MMP-9, TIMP-1; T-cell & monocyte cell activation, maturation, traffic phenotypes
Neural	NFL	t-tau, p-tau, Aβ_1-42_, sAPPα, sAPPβ

CCL2 (MCP-1, monocyte chemokine protein 1) a CSF marker for HIV and SIV encephalitis (Cinque et al. 1998; Kelder et al. 1998; Mankowski et al. 2004); CXCL10 (IP-10, interferon protein 10) likely the main chemokine for CSF lymphocytes (Cinque et al. 2005); beta-2-microglobulin (β2M), component of the MHC-I complex increased in CSF in HAD (Brew et al. 1992); TNFα (tumor necrosis factor alpha) also increased in the CSF in HAD (Mastroianni et al. 1990; Nolting et al. 2009); sCD14, the soluble LPS ligand increased in the CSF in HIV (Kamat et al. 2012); sCD163, a macrophage chemokine increased in blood in HIV (Kamat et al. 2012); sVCAM (soluble vascular adhesion molecule 1) mediates lymphocye and monocyte adhesion to vascular endothelium; MMP-9 (matrix metallopeptidase 9) and TIMP-1 (tissue inhibitor of metalloproteinases one) are involved in integrity of the extracellular matrix. T-tau (total tau) and p-tau (phosphorolated tau) are different states of the neuronal tau protein that are elevated in CSF in Alzheimer’s disease while amyloid beta 1–42 (Aβ1-42) is an amyloid cleavage product depressed in the CSF of Alzheimer’s disease (Blennow et al. 2010); sAPPα, sAPPβ are soluble degradation products of the amyloid precursor protein that are reduced in CSF in HAD (Gisslen et al. 2009)

Potentially informative CSF viral biomarkers include not only the concentration of HIV RNA using the same clinical methods applied to plasma, but also measurements of other features of the virus including the genetic relationship of CSF viral populations to those of plasma and differences in drug resistance, cell tropism and receptor usage between these two compartments (Spudich et al. 2005a; Schnell et al. 2010). At some future time, the list might also include more direct markers of neuropathogenicity, though beyond macrophage-tropism these have proved elusive (Dunfee et al. 2006). While the diagnostic value of CSF HIV RNA measurement is limited by detection in nearly all untreated individuals, the presence of virus and its drug resistance profile provides evidence and characterization of an active viral target. This evidence is essential to implicating CNS HIV infection as a possible cause of CNS disease and especially important in treated patients with neurosymptomatic viral escape (Canestri et al. 2010; Peluso et al. 2012) but perhaps also in other patients that harbor CNS resistance profiles different from those of plasma virus.

**Fig. 2:**
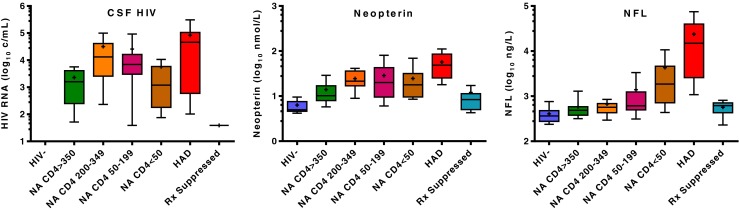
Changes in the three guide markers with disease progression and suppressive treatment studied cross-sectionally. The panels show the changes in CSF HIV RNA, neopterin and NFL with infection as systemic disease progresses, showing HIV uninfected controls (HIV-), neuroasymptomatic (NA) subjects with progressively lower blood CD4+ T cells, patients with HAD, and a group on suppressive therapy (*N* = 20 in each group except HAD with 12 and suppressed with 19). CSF HIV RNA was elevated in all untreated groups, and highest in HAD though in this small study only the treated-suppressed differed from the other infected groups (*P* < 0.05–<0.001). CSF neopterin was elevated in all the untreated HIV subjects, though highest in the HAD group (all HIV-infected groups differed from HIV- except CD4 > 350; HAD differed from suppressed and CD4 > 350; suppressed differed from all untreated except CD4 >350) (p values). NFL was highest in the HIV group (above normal in all) but NA subjects with lower CD4+ T cells showed a substantial prevalence of elevated levels indicative of subclinical CNS injury (HAD, CD4 <50 and 50–199 differed from HIV- while HAD differed from all groups except CD4 <50) (stat p values). Statistical comparison used Kruskal-Wallis test and Dunn’s post hoc test of multiple comparisons; graph and statistics prepared on Prism 6 (Graphpad Software Inc, San Diego, CA). *Box plots* show median and intraquartile range, ‘+’ shows mean and error bars the 10–90 percentiles

Biomarkers of immune and inflammatory responses include both soluble and cell-based markers. We have used neopterin, a pteridine biomarker metabolite that is readily measured in CSF (Hagberg et al. 2010), as our cardinal guide marker in this category (Angel et al. 2012). It is produced by cells of the monocyte-macrophage lineage and likely also by astrocytes (Cano et al. 2008) within the CNS compartment, reflects primarily stimulation by interferon gamma, and increases as systemic HIV infection progresses with highest levels in HAD (Fig. 2) and CNS opportunistic infections (Hagberg et al. 2010). Other soluble biomarkers include a range of cytokines, chemokines, markers of cell activation and blood-brain barrier dysfunction, a sample of which is listed in Table 1. CSF can also be used for cell-based assessment of T-cell and monocyte phenotypes related to activation, maturation and cell trafficking, though such studies are hampered by low cell numbers in CSF and the need for real-time assay, since CSF cells are fragile in storage (Ho et al. 2013). Since inflammatory responses and immunopathology are thought to be important in HIV neuropathology, markers in this class provide diagnostic evidence that disease is associated with inflammation (and thus plausibly caused by HIV) and may be helpful in following the effects of therapy (Yilmaz et al. 2013) Low-level residual elevation of CSF neopterin in treated patients also suggests that suppressive treatment does not always fully restore the normal CNS immunological milieu, though this low-level elevation of neopterin and other inflammatory biomarkers has not yet been clearly correlated with neurological progression (Gisslen et al. 2007b; Eden et al. 2010; Hagberg et al. 2010). Further delineation of the profile of inflammatory changes should provide further understanding of this central aspect of neuropathogenesis.

A number of neural biomarkers have been studied. We use NFL as a guide biomarker in this category (Abdulle et al. 2007), based on its sensitivity in detecting ongoing CNS injury in this setting, particularly when using the newer, more sensitive version of the assay available commercially (Krut et al. 2013; Peluso et al. 2013). Other neural markers that we and others have assessed are listed in Table 1 and include indicators of disturbed amyloid and tau metabolism (Clifford et al. 2009; Gisslen et al. 2009). Though not as sensitive as NFL, these biomarkers may be helpful in distinguishing HAD from Alzheimer’s disease. While none of these neural biomarkers are specific for HIV neuropathology, they importantly indicate active CNS injury in this setting (Gisslen et al. 2009). Glial markers can also be assessed, though they have not generally been found as useful (Andersson et al. 1998; Pemberton and Brew 2001).

While this discussion has focused on biomarker use in detecting or characterizing HIV-related CNS injury and omits consideration of biomarkers related to other neuropathogens (including particularly those causing opportunistic infections such as JC virus nucleic acid in progressive multifocal leukoencephalopathy or cryptococcal antigen in meningitis), it is important to emphasize the value of this class of diagnostic markers in neurological disease complicating HIV infection (Cinque et al. 2007). However, detailed discussion of these biomarkers is simply beyond the scope of this review.

## Choice of CSF as source of biomarkers and its limitations

Before more directly considering approaches to these measurements, it is appropriate to ask: Why focus on CSF? The main rationale for this rests on: 1) CSF’s proximity to the brain and spinal cord with which it exchanges molecular components, and thus allows sampling of the brain microenvironment (Yilmaz et al. 2012); and 2) its ready availability for sampling, including repeated sampling for longitudinal observation, with low morbidity (de Almeida et al. 2011). Importantly, while CNS HIV infection originates with viral and immune components of systemic infection transposed into the CNS, infection is variably ‘compartmentalized’ and features viral and immune components that are not clearly reflected in blood. Thus, the same measurements in CSF and blood may be dominated by disconnected or independent processes, and blood will not provide direct information about events within the nervous system.

Similarly, blood sampling would be a far more convenient approach to analysis of brain-derived neural biomarkers, but the obscuration, dilution or degradation of CNS metabolic traces in the blood have largely prevented blood from providing useful or direct information about brain pathobiology in HIV. This is, of course, also a major issue in other neurodegenerative diseases for which efforts are underway to develop sensitive assays that can measure informative molecules ‘spilling over’ from brain to blood. This has only yielded limited success thus far, particularly compared to the relative ease of such measurements in CSF (Blennow et al. 2010).

It is also pertinent to emphasize some of the biological pitfalls of interpreting CSF findings in HIV infection, particularly with regard to HIV and immune biomarkers. This relates to the mixed origin of these molecules *within the CNS* independent of systemic processes. Thus, CSF measurements may reflect processes localized within the leptomeninges, on the one hand, or within the brain and spinal cord parenchyma and associated perivascular spaces, on the other. Both of these anatomical CNS components are compartmentalized from blood and indeed they share constituents by diffusion. They may also be subject to parallel disease processes, including HIV infection and inflammation. However, they also may contribute differently to CSF sampled by lumbar puncture in different settings. At the extremes of evolving CNS HIV infection, CSF sampling can reflect predominantly meningeal or encephalitic infections, the first a nearly universal facet of untreated chronic infection and the second a late complication manifesting clinically as HAD. For this reason, interpretation of HIV and immune biomarkers must always ask whether a measured analyte derives from one or the other of these spaces (or even more directly, from blood). This is particularly at issue with viral and inflammatory biomarkers and less so with neural biomarkers that originate in the brain and not meninges, though spinal nerve roots may also potentially ‘contaminate’ CSF with neuronal breakdown products in the presence of neuropathies.

This distinction of compartment origin may be critical to interpretation. For example, HIV RNA can be measured in the CSF of nearly all untreated patients with detectable plasma viral loads, dating from the earliest sampling during acute infection through the chronic phase and in the presence of HAD with underlying HIVE (Ellis et al. 1997; McArthur et al. 1997; Spudich et al. 2005b, 2011; Valcour et al. 2012). Even in treated patients with plasma suppression, HIV RNA can be detected in CSF at very low levels using high fluid volumes and sensitive methods such as the single copy assay, though not as commonly as detection of such ‘residual’ virus in plasma (Dahl et al. 2011). This distinguishes detection of HIV RNA from most other meningitides or encephalidites in which detection of a virus in CSF proves CNS disease etiology (for example detection of CSF JC virus DNA in progressive multifocal leukoencephalopathy (Cinque et al. 2007). In HIV infection, the origin of virus detected in CSF changes across the spectrum of infection. In early infection the CSF HIV population is generally similar to that of blood and likely reflects origin in the meninges, production by trafficking CD4+ T cells and exhibiting T cell tropism (Arrildt et al. 2012; Swanstrom and Coffin 2012). However, it then becomes more compartmentalized, and this is particularly notable in HAD. While CSF virus populations in HAD may be either T cell- or macrophage (M)-tropic, their compartmentalization and CNS origin is demonstrated by the genetic distance from the blood population (Schnell et al. 2011). Because of these different viral sources, simply measuring the overall CSF HIV RNA level does not clearly indicate the origin or neuropathogenic effect.

The same difficulty can arise with some CSF immunological markers that reflect either meningeal inflammation, likely mainly originating or triggered by T cells, or parenchymal immune activation involving principally macrophages and related cells (Cinque et al. 1998; Hagberg et al. 2010; Burdo et al. 2013). While the component processes in these two spaces may diverge—potentially allowing dissection based on cytokine profiles – they also likely share some features that lead to overlap in some of the measured biomarkers, reducing their diagnostic and pathogenetic specificity.

## Applications of CSF biomarkers

There are two principal uses for CSF biomarkers: characterizing clinical states and defining pathogenesis. Though the same biomarkers may be used in both contexts, these objectives are sufficiently distinct that approaches to discovery and testing often differ.

### Clinical applications

Because of the current difficulties in clinical diagnosis and management of HIV-related CNS disease, CSF biomarkers promise to integrate objective, laboratory-based information into both clinical practice and clinical trials. Of particular importance is the capacity to segregate *active* disease from *static* injury with residual deficits. This is a crucial issue in patient evaluations and, more particularly, for treatment decisions. Both active and residual disease can present similar symptoms and signs along with similar phenotypes and severity of impairment on neuropsychological testing. However, only active virus-driven injury presents a target for direct intervention with initiation or modification of antiretroviral therapy or adjunctive measures targeting downstream effects. By contrast, static disease results from past injury with residual deficits which, while still important, requires focus on restorative efforts including rehabilitation and supportive measures. This distinction is important, not only for the individual patient in clinical practice, but in defining and evaluating subjects in clinical trials in which patients with static disease can markedly reduce the effect size and power of evaluating interventions designed to mitigate active HIV-driven injury. Mislabeling of patients with inactive disease also interferes with studies designed to characterize biological features of presumed active disease and its correlates.

While longitudinal clinical observations can also be used to distinguish active from static disease (and to validate the biomarker definitions of active versus static injury), an accurate longitudinal perspective is often difficult to obtain during a single clinic visit or at the start of a clinical trial where only a ‘snapshot’ is available. Biomarkers of active CNS disease potentially can also be used as surrogate clinical trial endpoints, much like plasma HIV RNA and blood CD4+ T cells are used in studies of systemic disease treatment. Obviously, these clinical applications require robust and reproducible analytical methods and established reference standards. Hence, initial approaches as emphasized in this review need to be followed by more rigorous validation studies so that a *pathogenetic biomarker* can transition to a valid *clinical surrogate marker* (Neaton et al. 2010).

### Pathogenesis studies

CSF biomarkers can also provide important insight into pathogenesis, measuring component biological processes that are not apparent either clinically or by other measurements, including blood studies or neuroimaging. Studies of pathogenesis may also focus on several biomarkers simultaneously, aiming to define not only their role in our association with an aspect of disease production but also their interactions. Hence, while clinical studies often strive for parsimony and the value of one or only a few measures, pathogenesis studies may look at a broader spectrum of biomarkers to understand different components of disease. Both novel and established biomarkers may provide insight into underlying mechanisms and how these mechanisms relate to different stages or manifestations of CNS HIV infection and resultant disease. Studies of pathogenesis may also seek to identify therapeutic targets beyond virus replication, including ‘adjuvant’ strategies that seek to mitigate components of pathogenesis other than simple reduction in infection.

## Approaches to biomarker evaluation

Approaches to biomarker studies can be classified on the basis of a number of features. Here we review two different approaches and illustrate these with two of our recent studies, one targeted and hypothesis-directed and the other using an untargeted discovery design (Table 2).

**Table 2:** Two examples of biomarker studies

Study approach	Study structure	Subject number	Sample number	Study organization	Analysis by subject group	Use of defined guide biomarkers	Biological stability
1. Hypothesis-directed evaluation	Cross-sectional	112 HIV + 20 HIV-	132	Observational cohort samples	Primary analysis	Secondary analysis	Static
2. Untargeted discovery	Longitudinal	14 HIV + 10 HIV-	91	Observational, ‘experimental’ intervention	Optimized by design using common baseline features, intervention and timing	Primary analysis	Dynamic

### Targeted biomarker evaluation

Our ongoing targeted, hypothesis-directed study is typical of an approach in which candidate biomarkers are examined for their possible clinical application (though also with a background interest in their pathogenetic implications). Biomarkers included in this type of study are often selected on the basis of blood biomarkers that might also be useful in CSF, observations made in cell culture or animal studies, or theoretical promise related to a particular facet of disease. Using data extracted from this study, we illustrate results measuring the three cardinal guide biomarkers listed earlier (Fig. 2). HIV RNA (Mellors et al. 1997; Riddler and Mellors 1997; Egger et al. 2002) and neopterin (Mildvan et al. 2005) were first used as blood biomarkers in HIV infection while CSF NFL has been applied as a biomarker of CNS injury in a variety of neurological conditions (Rosengren et al. 1996; Gunnarsson et al. 2011; Bech et al. 2012; Peluso et al. 2013).

As shown in Fig. 2, we assessed these CSF biomarkers in defined groups of patients representing phases of untreated systemic disease progression (patients without overt neurological symptoms divided into four groups by blood CD4+ T lymphocyte counts), a group with HAD, an antiretroviral treated group with plasma and CSF suppression and an HIV uninfected control group. The results shown here are similar to those previously published related to larger independent samples (Spudich et al. 2005b; Gisslen et al. 2007a; Hagberg et al. 2010). They show a rise in CSF HIV RNA with systemic progression, lower concentrations in patients with <50 blood CD4+ cells per μL, and the highest level in HAD. CSF neopterin was also elevated in all HIV-infected patients, with highest in HAD and reduction in the treated group, though perhaps not quite to HIV negative control levels (Yilmaz et al. 2008; Hagberg et al. 2010). CSF NFL showed the most distinct increase in HAD (elevated in all patients) but the concentration of this neural biomarker was also elevated in a substantial number of neuroasymptomatic with reduced blood CD4+ T cells, indicating subclinical neuro-axonal injury in these subjects (Gisslen et al. 2007a; Mellgren et al. 2007). The CD4+ T cell divisions used to define these groups not only have meaning with respect to systemic disease state, they are helpful in examining evolution of the CSF biomarker concentrations with progressive immunosuppression, and we are therefore using this subject-grouping to explore other biomarkers, including the ones listed in Table 1.

Because the biomarkers were defined at the initiation of study, they address an articulated or implied hypothesis—the association of the biomarker with a particular aspect of disease evolution. Typical of this type of study, it used a cross-sectional, observational structure drawing on archived samples from a relatively large number of subjects, though not as large as other more definitive studies. The results illustrate a number of important features of advancing disease as described in the legend of Fig. 2.

While the initial analytical approach compared the defined subject groups, analysis can also be extended to examine interrelations among the measurements across the larger subject sample. Analysis of one or a few novel biomarkers using multiplex assays is relatively straightforward at this exploratory level because of the circumscribed number of variables examined. This type of cross-sectional design can be supplemented by longitudinal studies to define the natural history and progression or response to therapy of individual subjects (Spudich et al. 2005b).

In undertaking these studies, we started with a simple classification of HIV-infected individuals, separating those with HAD from those without overt neurological symptoms and signs, listed as neuroasymptomatics (NA). This strategy, for the time being, ignores the important issue of using biomarkers to define milder CNS disease in untreated and treated subjects that has been classified under the Frascati criteria as minor neurocognitive disorder (MND) and asymptomatic neurocognitive impairment (ANI) defined by similar impairment levels on formal neuropsychological testing (Antinori et al. 2007). Along with HAD, these designations define the three levels of HIV-associated neurocognitive disorders (HAND).

In practice defining the biomarkers related to MND and ANI conditions presents a formidable problem. These conditions are etiologically and pathogenetically more heterogeneous and ambiguous (Heaton et al. 2011; Bonnet et al. 2013), and can be applied to patients with both active neurodegeneration and static residual injury from past insults, clearly different biological states. For this reason we, like other groups, have begun to explore biomarkers by defining HAD with the general idea that eventually one will be able to use these findings to ‘work backwards’ in exploring their application to the milder disease that is now of particular interest in treated populations in the developed world.

### Untargeted biomarker discovery

This illustrative second study was not limited by a priori hypotheses and used high-throughput proteomics, a discovery-based approach to identify novel protein biomarkers and identify their possible relationships (Angel et al. 2012). This, the first of our HIV CSF proteomic studies, used a different study structure and analyzed CSF from a much smaller group of subjects sampled longitudinally in the context of initiating antiretroviral therapy. For this study, we viewed the initiation of treatment as an ‘experimental’ intervention that rapidly and profoundly altered the infection and its inflammatory and neural consequences, leading to related changes in CSF biomarker concentrations. For some subjects we also included longitudinal observations of progressive untreated disease before treatment initiation. Part of the rationale for this approach related to the potentially obscuring effects of within-subject CSF proteome features that might not be relevant to the question being addressed. We reasoned that a smaller number of subjects with repeated sampling during a period treatment- or disease-related biological perturbation, could reduce the impact of these idiosyncratic subject effects, and, in fact, individual subjects did show distinctive features of the CSF proteome through the course of sampling (Angel et al. 2012).

However, using this approach how does one then analyze and interpret such a heterogeneous group of subjects with different baseline states, variable timing of and responses to ART and different number of samplings? For our initial analysis we used the three cardinal biomarkers listed earlier and examined how they correlated with features of the proteome over the entire sample set. This allowed us to identify proteins that varied in concentration with these markers, and thus were associated with changes in infection, immune activation-inflammation and neural injury as defined by these guides, thereby providing important context for the measured changes in these identified proteins and an initial description of their correlations and biological pathway relations. We also more directly examined correlations among the identified proteins themselves (Angel et al. 2012).

**Fig. 3:**
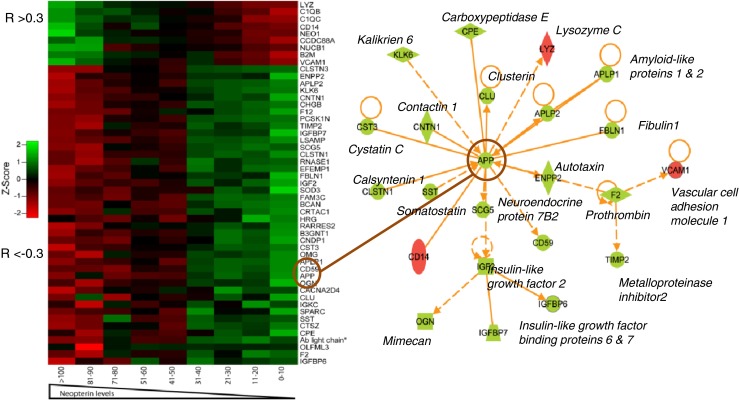
Protein correlations with CSF neopterin and pathway diagram. The heat map on the left-side diagrams the significant protein correlations (proteins with R values either >0.3 or < −0.3 by Spearman analysis) with decreasing concentrations of CSF neopterin. The *upper nine rows* show normalized (Z scores) protein concentration groups with positive correlations (*green to red*) while those below show negative correlations (*red to green*). The *right diagram* shows results of a pathway analysis that included the highest number of previously defined relationships of these neopterin correlating proteins and identified APP (amyloid precursor protein) as a ‘node’ in these relationships using Ingenuity Pathway Analysis (http://www.ingenuity.com/). Thus, using neopterin as an external biomarker we were able to identify an unrelated CSF neuronal protein that had previously shown to correlate with HAD, thus validating this overall approach with respect. The pathway analysis also suggests possible links in the pathogenesis related to these protein changes, based on previously reported interactions of the correlating proteins. Abbreviations use standard nomenclature. Undefined abbreviations on right panel include: *APP* amyloid precursor protein; *CD14* cluster of differentiation 14, also monocyte differentiation antigen that serves as lipopolysaccharide (LPS) co-receptor. Data are from Angel et al. (Angel et al. 2012) where the findings are described in greater detail

Among the differences in this type of discovery study and the hypothesis-based study as described above, is that the large number of identified proteins present a formidable informatics and analytical challenge. The use of the guide biomarkers allows an initial view of the proteome that can then be extended to an increasingly broad perspective by building on these associations. Thus, these initially defined endophenotypes serve as a scaffold for extended explorations. Figure 3 shows an example of this from our proteomics study, listing correlating proteins and pathway diagram incorporating several of these (Angel et al. 2012).

Cross-sectional design can also be used for pathogenetic studies, and indeed the cross-sectional study described earlier represents one phase of a broader study that began with definitions of guide biomarkers, has been extending to additional predefined biomarkers, and is now being further extended through proteomic analysis. In this study, differences in the proteome between groups and across the entire sample can be analyzed in relation to these defined guide biomarkers.

Once this type of study reveals a series of pathogenetically interesting proteins, the challenges then include: ‘validating’ the general findings using more quantitative assays, most often immunoassays, applied to some of the salient proteins; reproducing the salient findings independently using an independent sample set or experimental paradigm; and finally beginning to explore causality in the various relationships suggested by the correlations and pathways. In this sense, this type of exploratory methodology is only the beginning of pathogenetic exploration, but nonetheless invaluable. Similarly, identified proteins that appear attractive as clinical biomarkers need to be characterized further using simpler, more quantitative and eventually reproducible assay methods that can be broadly applied in clinical settings.

## Classification of subjects and samples

Whatever approach is taken to characterize CSF biomarkers in HIV infection, a critical requirement is the careful and clear classification of study subjects and samples. This is a common feature of both hypothesis-driven and discovery methods. There is more than one structural approach to this issue. This includes the use of biologically meaningful, well-defined subject groups for cross-sectional analyses as illustrated above with the subject divisions across the spectrum of systemic progression, transition to HAD and treatment. Longitudinal studies may also rely on patient classifications, but also can benefit from a defined structure that includes similar baseline characteristics, interventions and scheduled assessments that allow subject or time groupings for analysis. The second approach, using independent biomarkers to classify samples, is generally more flexible and can be used for either primary or secondary analysis.

**Fig. 4:**
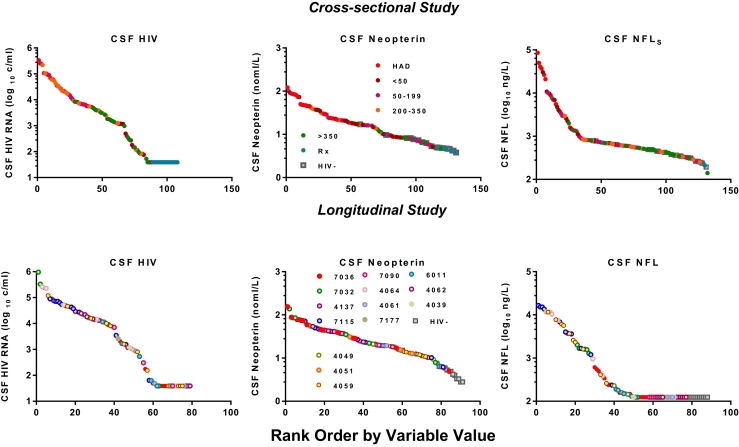
Comparison of biomarker sample distributions of two studies. In each panel the sample results have been sorted by value from highest to lowest for the listed biomarkers. The top three panels show data from the cross-sectional study outlined earlier and presented by group in Fig. 2 using the same color scheme that is also defined in the middle panel. The lower three panels show sorted samples from the longitudinal study, with those from each individual subject now identified by color as shown in the middle panel. The X axes differ because of the larger number of samples in the cross-sectional study. The CSF HIV RNA (left panels) shows a similar distribution in the two studies from the level of quantitation (40 copies per mL) to >100,000 copies per mL. Similarly, the CSF neopterin concentrations of both sample sets span a similar distribution. The CSF NFL values in the two studies show differences that relate to two factors. First, the assays differed in the two studies with the cross-sectional study (actually performed more recently) using the newer, more sensitive assay (Peluso et al. 2013) accounting for both the higher values and the continued value spread at the lower end of concentrations (including within the normal value distribution below 890 ng per L), whereas in the second study the lower half of the sample was below the detection limit of 125 ng/L) for the older assay (Gisslen et al. 2007a). Second, the longitudinal study had a larger number of samples from neurologically abnormal subjects as a result of selection of HAD patients and those with low blood CD4+ cells for study. Overall, both studies show a continuous distribution of values for each of the CSF biomarkers

Figure 4 compares some of the sample features of the two illustrated studies, examining the distributions of concentrations of the guide biomarker. Both designs provided sample sets that spanned a wide and biologically meaningful spectrum of concentrations for all three of the measurements. This is an important aspect of sample selection since it predicts a similar range for proteomic or other analyses and for defining associations among the biomarkers. The differences in the sample sets are described in the figure legend. In the cross-sectional study, the specimen spectrum was composed of independent subjects presumably at a relatively steady state at the time of sampling, since all were clinically and virologically stable at sampling. In the longitudinal study, a smaller group of subjects assume interdigitating places across the spectrum of concentrations. Possible issues with this second approach include the variable number of samples for individual subjects and the dynamic state of the patients at many of these points. On the other hand, this dynamic state may be important for biomarker associations as well in measuring their changes with treatment. The two studies can be regarded as complementary in several aspects and together should provide both a steady-state and dynamic view of CSF biomarker changes.

## Conclusions

Despite the early recognition of the importance of CNS HIV infection and its link to brain injury, diagnosis and management has been limited by the lack of objective measures of disease. While CSF biomarkers have already been useful in defining the natural history of these linked processes and their responses to treatment, they have been underexploited for pathogenetic study and have found very limited use in the clinic for either patient management or clinical trials. While there is clear need for further characterization of already-studied biomarkers and discovery of additional useful biomarkers, it is also time to undertake studies that validate available CSF biomarkers and open the way for their use in clinical practice either as single markers or using a combinatorial approach (Gisslen et al. 2007b).
